# Detection of cofilin mRNA by hybridization-sensitive double-stranded fluorescent probes†

**DOI:** 10.1039/c7ra13349a

**Published:** 2018-02-16

**Authors:** Ha Jung Lee, Gui Han Go, Jong Jin Ro, Byeang Hyean Kim

**Affiliations:** Department of Chemistry, Division of Advanced Materials Science, Pohang University of Science and Technology (POSTECH) Pohang 37673 Republic of Korea bhkim@postech.ac.kr

## Abstract

We have developed hybridization-sensitive fluorescent oligonucleotide probes that, in the presence of quencher strands, undergo efficient fluorescence quenching through the formation of partial DNA/DNA duplexes. In the presence of target RNA, rapid displacement of the quencher strands results in highly enhanced fluorescence.

## Abstract 2

We have developed hybridization-sensitive fluorescent oligonucleotide probes that, in the presence of quencher strands, undergo efficient fluorescence quenching through the formation of partial DNA/DNA duplexes.

## Graphical abstract

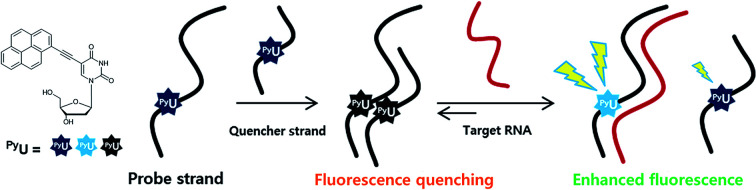

The detection of biomolecules is an important part of any investigation into their biological mechanisms and phenomena. Fluorescence-based methods are particularly useful for providing interpretable signals for various targets (*e.g.*, genes, proteins, small molecules). When nucleic acids are used as probes, they can provide sequence-specific information regarding the binding (through hydrogen bonding) of target nucleic acids. Because of their high sequence-specificity, many fluorescent hybridization probes, including molecular beacons (MBs), have been developed and applied for nucleic acid detection and visualization.^1,2^

In previous studies, we found that a quencher-free molecular beacon (QF-MB) containing the pyrene-modified nucleoside ^Py^U exhibited a high fluorescence enhancement in the presence of trinucleotide repeats, especially for RNA.^3,4^ Among various fluorescent nucleobase derivatives, uracil derivatives have been particularly useful for selective detection of specific sequences, taking advantage of changes in photoinduced electron transfer between the fluorophore and the neighboring base.^3–5^ To apply such systems to various other target sequences, here we designed fully complementary sequences and incorporated the internal fluorescent nucleoside ^Py^U in place of a thymine residue, resulting in a significantly increased fluorescence signal based on strand displacement (Fig. 1). Incorporation of a ^Py^U unit in a single strand of the probe sequence and hybridization with a strand partially complementary to the probe strand containing a pyrene unit as a fluorescence quencher can lead to improved discrimination factors.^4,6^ Such partially double-stranded probes have several attractive features.

**Fig. 1:**
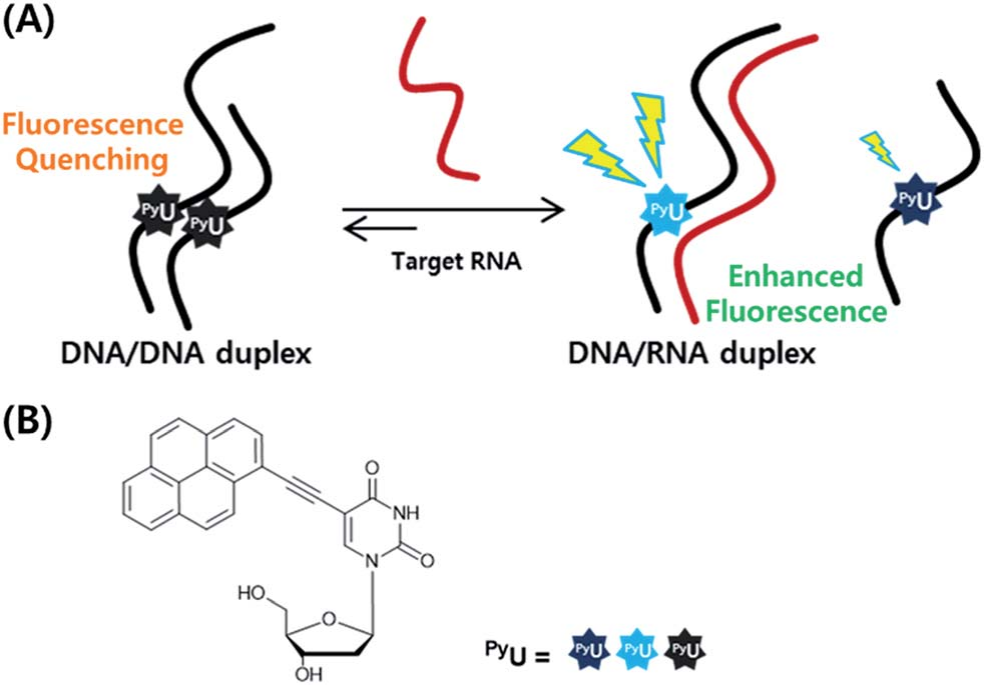
(A) Schematic representation of the strand displacement process developed in this present system. (B) Structures of the internal fluorophore ^Py^U.

First, the probe sequence does not require an additional sequence in its strand that is not complementary to the target sequence to ensure formation of a secondary structure (*e.g.*, a hairpin). Such additional sequences might interfere with the specific hybridization between the probe and the target sequence. Accordingly, double-stranded fluorescence probes should allow the specific detection of many kinds of targets. Second, the highly quenched initial fluorescence signal, due to the formation of a partial duplex, results in significantly increased fluorescence in the presence of the target; the incorporation of a hybridization-sensitive internal fluorophore provides a stable and sensitive fluorescence signal upon perfect hybridization with the target.

Cofilin is a protein that regulates the activity of actin, which is related to the formation of the cytoskeleton in cells. Actin plays a crucial role in the growth and elongation of cells,^7^ especially in neurons and, therefore, in the control of neurotransmission. We designed probe strands complementary to the 3′-untranslated region (3′-UTR) of target cofilin mRNA and synthesized three kinds of 19-*mer* probe strands (P1–P3) containing one or two ^Py^U units in each strand (Table 1). We measured the absorption and fluorescence emission spectra of these probes as both single strands and as double strands with target RNA (Fig. 2A and S1, ESI†). The fluorescence intensities of P1 and P3 increased dramatically after binding with the target RNA T19—by 17.6- and 16.0-fold, respectively. For the probe P2 (in which the ^Py^U residue was located close to the 3′-end), however, the fluorescence enhancement was very low: only 1.8-fold (Table S2, ESI†). We assume that terminal modification of the ^Py^U unit in the probe resulted in weak base pairs around the ^Py^U residue than did central modification, resulting in a decrease in fluorescence enhancement (Table S3, ESI†).^8^ Because flanking base pairs around the ^Py^U unit are relatively less rigid compared to central base pairs, the microenvironment of ^Py^U in the duplex formed from P2 and T19 is different from the central modification. Therefore, the fluorescence intensity did not increase significantly compared to the single-stranded P2. The absorption spectrum of P2 in the presence of T19 exhibited a relatively less intense absorption band than that of the duplex formed from P1 and T19—the latter featured an intense signal corresponding to the high fluorescence intensity (Fig. S1, ESI†).^9,10^ Even though two ^Py^U units were incorporated into the single strand P3, its fluorescence enhancement was similar to that of probe P1 containing only one ^Py^U unit. Among other examined probe sequences complementary to other parts of 3′-UTR in cofilin mRNA, P6 (containing two ^Py^U units) also exhibited fluorescence intensity similar to those of P4 and P5, single-^Py^U – containing probes each modified in the central position (Fig. S2, ESI†). These results suggest that single modification of a ^Py^U unit in the probe sequence is more efficient than dual modification, in terms of inducing high fluorescence enhancement with target RNA.

**Table 1:** Oligonucleotide sequences of probe and target strands

Name	Sequence
P1	5′-GGT GCC ^**Py**^**U**AG GAC GGG ACT T-3′
P2	5′-GGT GCC TAG GAC GGG AC^**Py**^**U** T-3′
P3	5′-GGT GCC ^**Py**^**U**AG GAC GGG AC^**Py**^**U** T-3′
U5	3′-CA CGG ^**Py**^**U**TC CTG-5′
U6	3′-CCA CGG ^**Py**^**U**TC CTG C-5′
T19^a^	5′-a agu ccc guc cua ggc acc-3′
T19-U^a^^,^^b^	5′-a agu ccc guc cu*u̲* ggc acc-3′
T19-G^a^^,^^b^	5′-a agu ccc guc cu*g̲* ggc acc-3′
T19-C^a^^,^^b^	5′-a agu ccc guc cu*c̲* ggc acc-3′

a Target RNA sequence.

b Underlined letter indicates a single mismatched base.

Next, to improve the fluorescence enhancement in the presence of the target, the background signal of the probe was decreased by mixing it with a pyrene-modified short oligonucleotide, a so-called “quencher strand”, capable of quenching the fluorescence of the ^Py^U unit. Two pyrene units on the opposite side in the duplex resulted in fluorescence quenching because pyrene moieties are stacked each other and located in a highly polar environment.^4,9^ Such duplexes containing probe and quencher strands would have to undergo displacement of the quencher strand prior to hybridization of the target strand. First, we tested the effects of a ^Py^U residue located in the quencher strand at the central position, opposite the ^Py^U residue in the probe strand, potentially minimizing the fluorescence of the ^Py^U residue in the probe strand through π-stacking of the two pyrene units in the duplex.^4,11^ We synthesized the quencher strands U5 and U6, each containing a ^Py^U residue, and examined the relationship between the quenching efficiency and stable hybridization of the probe/quencher duplexes upon varying the length of the quencher strand (Table 1). The fluorescence intensity of the probe was indeed affected by the length of the quencher strand (Fig. 2B). The more stable the partial duplex is formed, the more effective stacking interaction between pyrene moieties close to each other can be possible.^12^ In the presence of the ^Py^U-modified quencher strands, the quenching efficiency of the probe at 435 nm was 65% for U5 and 80% for U6. As a result, the enhancements in fluorescence for the probe in the presence of the target T19 were 51.5- and 66.7-fold for U5 and U6, respectively (Fig. 2C and S7, ESI†) much higher than that for P1 alone. Notably, these fluorescence signals were generated not only from the probe/target duplexes but also from the released quencher strands (*i.e.*, the single-stranded quencher strands also exhibited fluorescence to some degree). Therefore, the actual fluorescence signal arising from hybridization of the probe with the target was slightly lower than that observed in the fluorescence spectra; we estimated that the additional signals due to the release of U5 and U6 increased the fluorescence intensity by 7% (Fig. S7, ESI†). In other words, the released quencher strands added to the fluorescence enhancement of the DNA/RNA duplex. Moreover, we also tested the effect of incorporating a dabcyl derivative, ^Dab^U, as a typical fluorescence quencher on the quencher strand and compared its effects with those of the quencher strand containing a ^Py^U unit (Table S4, Fig. S4, ESI†). The ^Py^U-modified quencher strands provided the probe with similar quenching in fluorescence as did ^Dab^U-modified quencher strands of the same length (Fig. S8 and S9, ESI†). The melting temperatures (Fig. S10, Table S5, ESI†) of the duplexes of the ^Py^U-modified quencher strands and P1 (for U5 and U6 with P1: 60.1 and 66.0 °C, respectively) were higher than those of the natural strands and P1 (for N5 and N6 with P1: 46.8 and 56.0 °C, respectively); the former were stabilized through π-stacking of the ^Py^U units (Fig. S5 and S6, ESI†). The formation of duplexes from the probe and quencher strands was evident also in circular dichroism (CD) and polyacrylamide gel electrophoresis (PAGE) experiments (Fig. S11 and S12, ESI†).

**Fig. 2:**
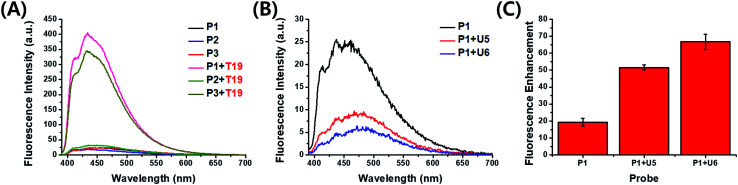
(A, B) Fluorescence emission spectra of (A) the probes P1–P3 with T19 and (B) the probe P1 with U5 and U6. (C) Fluorescence enhancements (*F*/*F*_0_) at 432 nm of P1 in the presence of U5 and U6 upon binding with T19; 1.0 μM of samples in 100 mM Tris-HCl buffer (pH 7.2), 100 mM NaCl and 10 mM MgCl_2_; annealing: 90 °C; excitation wavelength: 380 nm; excitation/emission slit: 5 nm/5 nm; *F*: fluorescence intensity at 432 nm of the probe P1 with the target RNA T19 in the absence or presence of a quencher strand U5 and U6; *F*_0_: fluorescence intensity at 432 nm of the probe P1 in the absence or presence of a quencher strand U5 and U6.

To confirm the effective strand displacement of the quencher strand from the probe strand, we conducted time-dependent fluorescence experiments after addition of the target RNA T19 to probe/quencher duplexes (Fig. 3). After addition of the target strand to the single strand of P1, hybridization was complete within 30 min (*i.e.*, the increase in fluorescence at 435 nm was minor thereafter). In contrast, the fluorescence intensity of P1 in the presence of the 11-*mer* quencher strand U5 was relatively rapid, reaching equilibrium after 20 min; for the 13-*mer* strand U6, however, equilibrium was reached within 35 min. Thus, compared with the single-stranded probe P1, the hybrid of P1 with U5 reacted more rapidly with the target T19, but the reaction time of the hybrid of P1 with U6 responding to the target T19 was slightly slower than that of P1 in the absence of a quencher strand. We suspect that the probe strand in the absence of a quencher strand was stabilized by stacking of the nucleobases; the probe would take some time to hybridize with the target RNA, requiring unfolding of its stacked bases. For the partially hybridized duplexes, however, the non-bonded sequence of the probe would be exposed, facilitating hybridization with the target strand. As a result, the response of P1 in the presence of U5 toward the target RNA was slightly faster than that of single-stranded P1 alone. Thus, as the length of the non-bonding sequence of P1 in the partial duplex decreased by increasing the length of quencher strand, the rate of strand displacement decreased accordingly.^13–16^ Indeed, the reaction time for the probe strand in the presence of the 15-*mer* strand U7 was much longer than those in the presence of the 11- and 13-*mer* quencher strands, because only a four-nucleotide sequence was available for hybridization of the target stand T19 (Fig. S13, ESI†); in addition, the equilibrium of the reaction shifted to the left, in conjunction with a smaller enhancement in fluorescence.

**Fig. 3:**
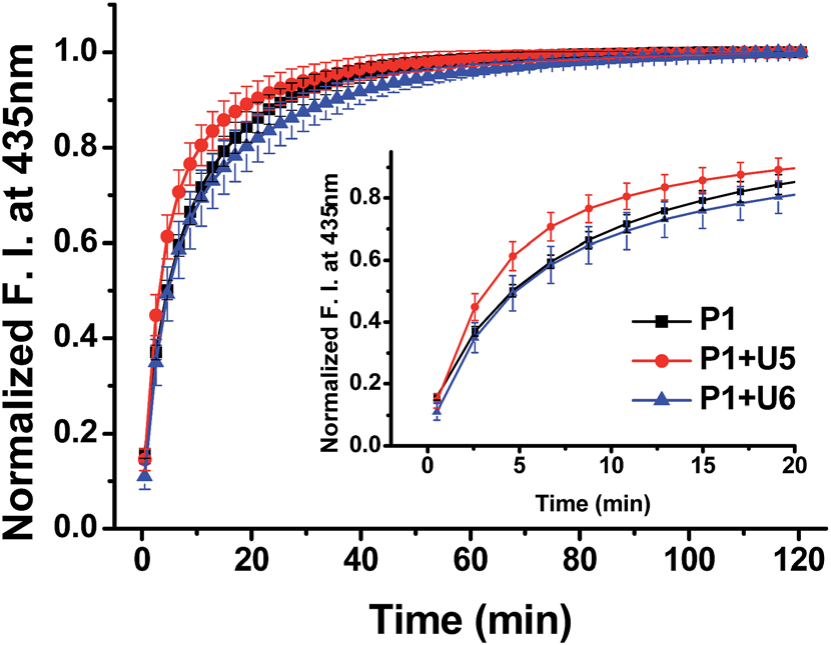
Time-dependent fluorescence of P1 in the presence of U5 and U6 after addition of T19; 1.0 μM of samples in 100 mM Tris-HCl buffer (pH 7.2), 100 mM NaCl and 10 mM MgCl_2_; excitation wavelength: 380 nm; emission wavelength: 435 nm; excitation/emission slit: 5 nm/5 nm; temperature: 20 °C.

We also tested the selectivity of the probe P1 itself against single-base-mismatched target RNA (Fig. 4, Table 1). Target RNA strands with mismatched bases opposite the ^Py^U residue in the probe strand provided no enhancements in fluorescence when the mismatched bases were uracil and cytosine. When guanine was the mismatched base, however, the presence of the target RNA led to a slightly increased signal. These fluorescence spectra suggest that the probe P1 is capable of selective fluorescence enhancements that can distinguish single-base-mismatched target RNA.

**Fig. 4:**
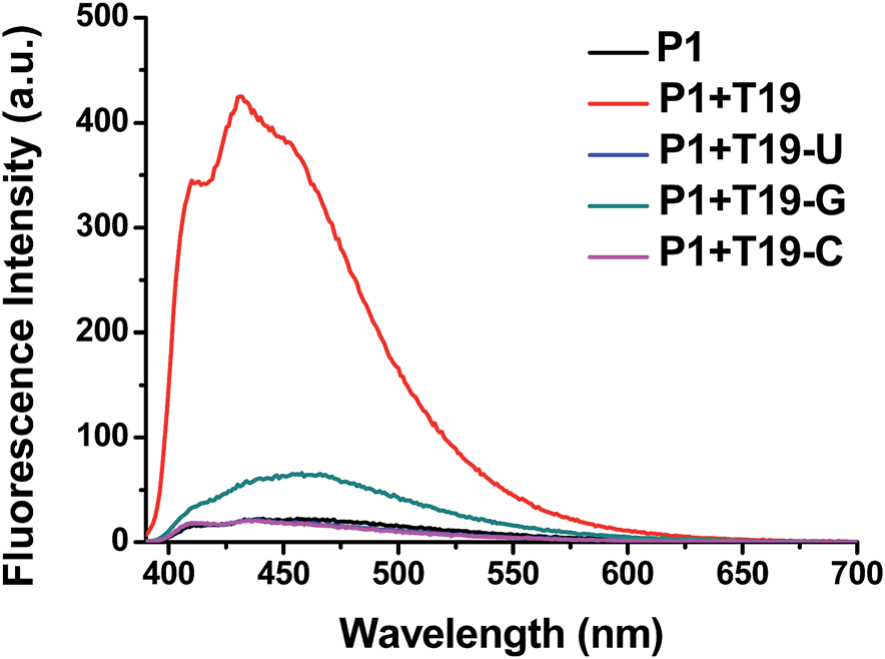
Fluorescence emission spectra of P1 in the presence of single-base-mismatched target RNA; 1.0 μM of sample in 100 mM Tris-HCl buffer (pH 7.2), 100 mM NaCl and 10 mM MgCl_2_; annealing: 90 °C; excitation wavelength: 380 nm; excitation/emission slit: 5 nm/5 nm.

In conclusion, we have developed a double-stranded duplex that functions as a universal probe that is highly specific for the sequence of its target RNA—in this case, for cofilin mRNA. When the ^Py^U-modified probe strand was partially hybridized with quencher strands containing a ^Py^U unit, the fluorescence intensity decreased dramatically as a result of π-stacking of the ^Py^U units. The probe/quencher hybrids provided even greater fluorescence enhancements after stable binding of the target RNA strand with the additional signal from the released quencher strand further improving the fluorescence detection of the target RNA.

## Conflicts of interest

There are no conflicts to declare.

## Supplementary Material

RA-008-C7RA13349A-s001
