# Supplementary material for: Detection of cofilin mRNA by hybridization-sensitive double-stranded fluorescent probes
Source: RSC Adv. 2018 Feb 16;8(14):7514–7. doi: 10.1039/c7ra13349a (PMC9078427; doi:10.1039/c7ra13349a)

Electronic Supplementary Material (ESI) for RSC Advances.
This journal is © The Royal Society of Chemistry 2018

Supporting Information

Detection of cofilin mRNA with hybridization-sensitive double-stranded fluorescent probes

Ha Jung Lee, Gui Han Go, Jong Jin Ro and Byeang Hyean Kim*

Department of Chemistry, Division of Advanced Materials Science, Pohang University of Science and Technology (POSTECH), Pohang 37673, Republic of Korea

Contents

S1 Experimental details

S2 **Table S1.** Probe sequences, containing ^{Py}U units, for each target sequence

S3 **Figure S1.** UV absorption spectra of **P1**–**P6** in the absence and presence of the target RNA

S4 **Figure S2.** Fluorescence emission spectra of **P4**–**P6** in the absence and presence of the target **T21**

S5 **Table S2.** Fluorescence enhancements of the probe sequences for the target RNA

S6 **Figure S3.** Melting curves of **P1**–**P3** in the presence of **T19** and **P4**–**P6** in the presence of **T21**

S7 **Table S3.** Melting temperature of each probe with its target RNA

S8 **Table S4.** Quencher sequences, containing a ^{Py}U or ^{Dab}U unit, partially complementary to **P1**

S9 **Figure S4.** Structure of the internal quencher ^{Dab}U

S10 **Figure S5.** UV absorption spectra and normalized UV absorption spectra of **P1** in the presence of **U5**–**U7**

S11 **Figure S6.** Temperature-dependent absorption spectra of single-stranded **P1** and **P1** in the presence of a quencher strand **U5**–**U6**

S12 **Figure S7.** Fluorescence emission spectra of single-stranded **P1** and **U5**–**U7** and **P1** in the presence of a quencher strand **U5**–**U7** and **T19**

S13 **Figure S8.** UV absorption spectra of **P1** in the presence of **Q5**–**Q7**

S14 **Figure S9.** Fluorescence emission spectra of **P1** in the presence of **Q5**–**Q7** and **T19**

S15 **Figure S10.** Melting curves of **P1** in the presence of **U5**–**U7** and **Q5**–**Q7**

S16 **Table S5.** Melting temperature of **P1** in the presence of **U5**–**U7** and **Q5**–**Q7**

S17 **Figure S11.** CD spectra of **P1** and **T19** and **P1** in the presence of a quencher strand **U5**–**U7** and **T19**

S18 **Figure S12.** Native polyacrylamide gel electrophoresis (PAGE) images of **P1** with **T19** in the presence of **U5**–**U7**

S19 **Figure S13.** Time-dependent fluorescence intensity of **P1** in the presence of **U7** after the addition of **T19**

Experimental details

Synthesis of oligonucleotides

^{Py}U- and ^{Dab}U-modified oligonucleotides (ODNs) were synthesized on a CPG support (1 μmol scale, 1000 Å pore size) using standard phosphoramidite methods and an automated DNA synthesizer (POLYGEN DNA-Synthesizer). The synthesized ODNs were cleaved from the solid support upon treatment with 28–30% aqueous NH_4OH (1.0 mL) for 12 h at 55 °C. After filtration of the CPG, the crude products from the automated ODN synthesis were lyophilized and diluted with distilled water (1 mL). The ODNs were purified through reverse-phase HPLC (Merck LichoCART C18 column; 10 × 250 mm; 10 μm; pore size: 100 Å). The HPLC mobile phase was held isocratically for 10 min with 5% MeCN/0.1 M triethylammonium acetate (TEAA) (pH 7.2) at a flow rate of 2.5 mL/min. The gradient was then increased linearly over 10 min from 5 to 50% MeCN/0.1 M TEAA at the same flow rate. The fractions containing the purified ODNs were cooled and lyophilized. 80% Aqueous AcOH was added to the ODNs. After 1 h at ambient temperature, the AcOH was evaporated under reduced pressure. The residue was diluted with water (1 mL); this solution was then purified through HPLC using the same conditions as those described above. The ODNs were analyzed through reverse-phase HPLC using almost the same eluent system (detection: 254 nm). The products were characterized using MALDI-TOF mass spectrometry.

ODN sample preparation for experiments

For UV spectroscopy, fluorescence spectroscopy, and melting temperature (T_m) and circular dichroism (CD) measurements, 1.0 (UV and fluorescence spectroscopy), 2.0 (T_m), and 3.0 (CD) μM of the ODN was added to a solution of 1 M Tris-HCl buffer (pH 7.2, 100 μL), 1 M NaCl (100 μL), 200 mM $MgCl_2$ (50 μL) and water (in a 1.5-mL microtube) to give a total volume of 1 mL, followed by vortex-mixing. In the case of duplexes, the probe ODN and the target RNA and/or the quencher ODN were added to 1 M Tris-HCl buffer (pH 7.2, 100 μL), 1 M NaCl (100 μL), 200 mM $MgCl_2$ (50 μL) and water and then the sample subjected to vortex-mixing. To prepare annealed samples, the mixtures in a buffer solution were heated at 90 °C for 3 min, then slowly cooled under ambient conditions for 4 h.

UV and fluorescence spectra

UV and fluorescence spectra were recorded using Cary 100 and Eclipse spectrometers (Varian), respectively. Samples for UV and fluorescence spectroscopy were prepared in a quartz cell (path length: 1 cm). All samples were measured after baseline correction for UV spectra. Parameters for fluorescence spectra; excitation wavelength: 380 nm, scanning range: 390–700 nm, excitation and emission slits: 5 nm/5 nm; data interval: 1.0 nm.

Melting temperatures (T_m)

All values of T_m were recorded at 260 nm in a quartz cell (path length: 1 cm) using a Cary 100 Conc UV–Vis spectrophotometer (Varian) equipped with a temperature controller. The values of T_m were calculated from the maximum values of the first derivatives in plots of absorbance with respect to temperature.

CD spectroscopy

CD spectra of the ODNs were recorded using a J-810 apparatus (JASCO) equipped with a temperature controller. For each sample, five spectral scans were accumulated at 20 °C over the wavelength range from 220 to 340 nm.

20% Native polyacrylamide gel electrophoresis (PAGE)

5 mL of 40% acrylamide, 2 mL of 5X TBE buffer, and 3 mL of distilled water were mixed for 20% non-denaturing gel. 12 mg of ammonium persulfate was added to the mixed solution. For initiation of gel formation, 10 μL of *N*, *N*, *N*′, *N*′-tetramethylethylenediamine (TEMED) was added. 200 pmol of samples were used for PAGE. Dried samples were dissolved in 10 μL of buffer/formamide mixture (v/v = 1 : 1) for sample loading. PAGE was carried out at 90 V, 26 mA, 3 W, 25 °C for 2.5 h. After running, gels were mixed with stains all (Sigma-Aldrich) in formamide for 30 min. Gels were dried and exposed on light for visualization of DNA bands.

Table S1. Probe sequences, containing ^{Py}U units, for each target sequence

Target RNA sequences

T19 (19-mer): 5′-a agu ccc guc cua ggc acc-3′

T21 (21-mer): 5′-uga cca cuc aug gaa gca gga-3′

Natural DNA sequence

PN (19-mer): 5′-GGT GCC TAG GAC GGG ACT T-3′

**Name**	**Sequence**	**Calculated MS (*m/z*)**	**Observed MS (*m/z*)**
**P1**	5′-GGT GCC ^{Py}**U**AG GAC GGG ACT T-3′	6092.0637	6092.4612
**P2**	5′-GGT GCC TAG GAC GGG AC^{Py}**U** T-3′	6092.0637	6092.3920
**P3**	5′-GGT GCC ^{Py}**U**AG GAC GGG AC^{Py}**U** T-3′	6302.1106	6301.6128
**P4**	5′-TCC ^{Py}**U**GC TTC CAT GAG TGG TCA-3′	6595.1380	6595.6727
**P5**	5′-TCC TGC ^{Py}**U**TC CAT GAG TGG TCA-3′	6595.1380	6594.1879
**P6**	5′-TCC ^{Py}**U**GC ^{Py}**U**TC CAT GAG TGG TCA-3′	6805.1849	6805.3771

Figure S1. UV absorption spectra of **P1**–**P6** in the absence and presence of the target RNA

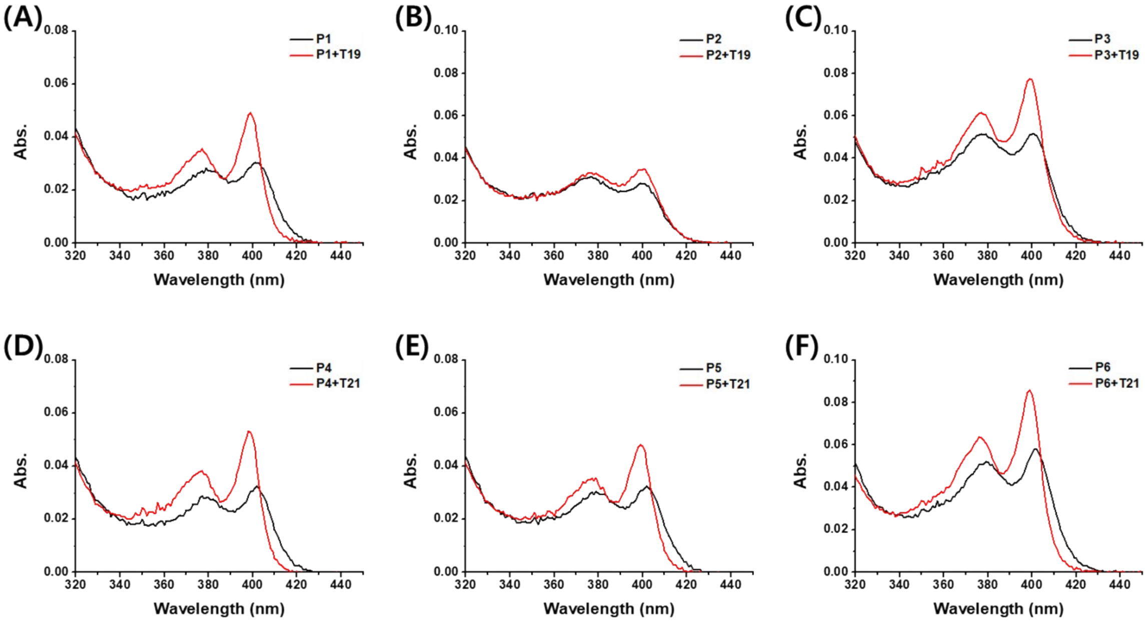

Figure S2. Fluorescence emission spectra of **P4–P6** in the absence and presence of the target **T21**

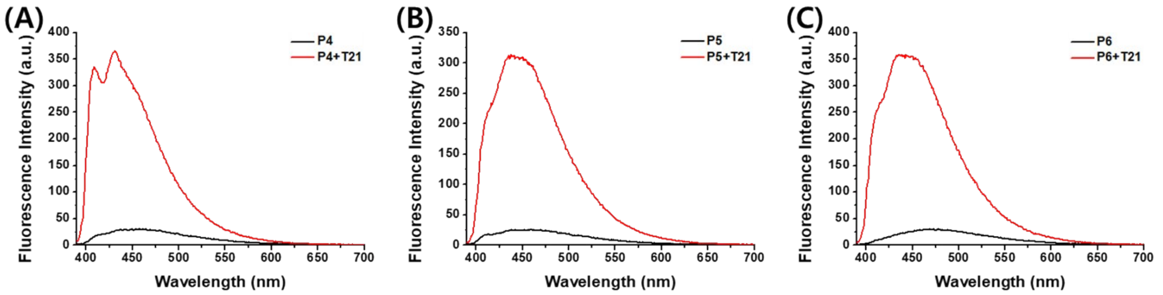

Table S2. Fluorescence enhancements of the probe sequences for the target RNA

	Wavelength (nm)	Fluorescence enhancement
**P1**	434	17.6
**P2**	435	1.8
**P3**	433	16.0
**P4**	431	13.6
**P5**	438	12.5
**P6**	439	16.2

Figure S3. Melting curves of (A) **P1–P3** in the presence of **T19** and (B) **P4–P6** in the presence of **T21**

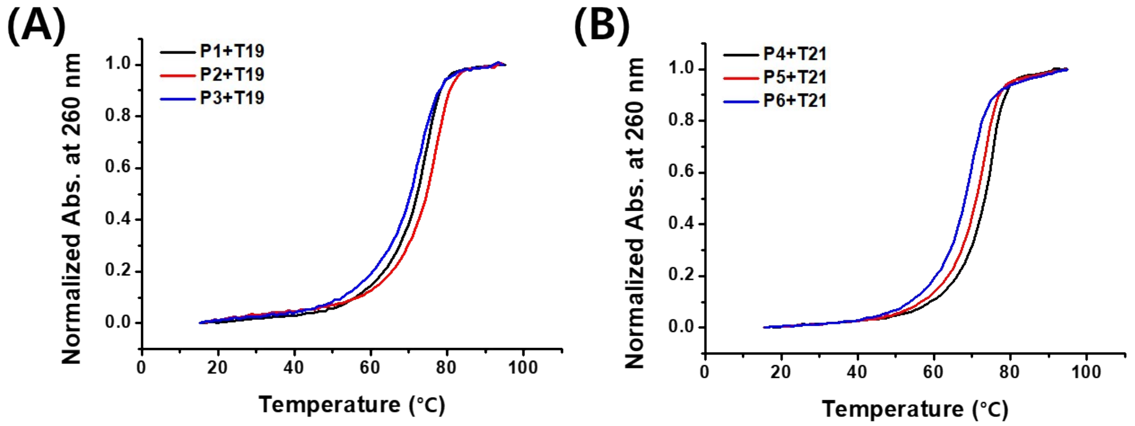

Table S3. Melting temperature of each probe with its target RNA

Duplex	T_m (°C)
**PN + T19**	74.7
**P1 + T19**	71.4
**P2 + T19**	74.4
**P3 + T19**	70.1
**P4 + T21**	72.8
**P5 + T21**	70.6
**P6 + T21**	67.7

Table S4. Quencher sequences, containing a ^{Py}U or ^{Dab}U unit, partially complementary to **P1**

Natural DNA sequences

N5: 3´-CA CGG ATC CTG-5´

N6: 3´-CCA CGG ATC CTG C-5´

N7: 3´-CCA CGG ATC CTG CCC-5´

Name	Sequence	Calculated MS (*m/z*)	Observed MS (*m/z*)
**U5**	3´-CA CGG ^{Py}**U**TC CTG-5´	3516.6354	3516.9267
**U6**	3´-CCA CGG ^{Py}**U**TC CTG C-5´	4094.7292	4093.9226
**U7**	3´-CCA CGG ^{Py}**U**TC CTG CCC-5´	4672.8230	4672.1072
**Q5**	3´-CA CGG ^{Dab}**U**TC CTG-5´	3539.6838	3539.4791
**Q6**	3´-CCA CGG ^{Dab}**U**TC CTG C-5´	4117.7776	4118.6337
**Q7**	3´-CCA CGG ^{Dab}**U**TC CTG CCC-5´	4695.8714	4695.1693

Figure S4. Structure of the internal quencher ^{Dab}U

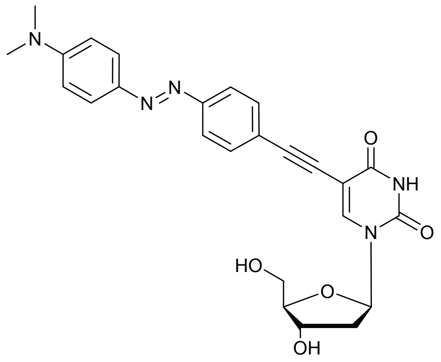

^{Dab}U

Figure S5. (A) UV absorption spectra and (B) normalized UV absorption spectra of **P1** in the presence of **U5**–**U7**

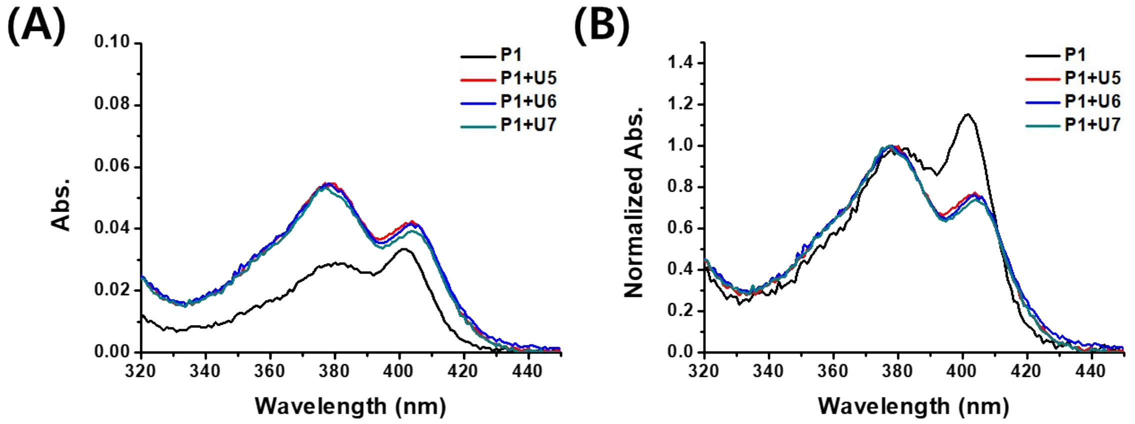

Figure S6. Temperature-dependent absorption spectra of (A) single-stranded **P1** and (B, C) **P1** in the presence of a quencher strand **U5**–**U6**

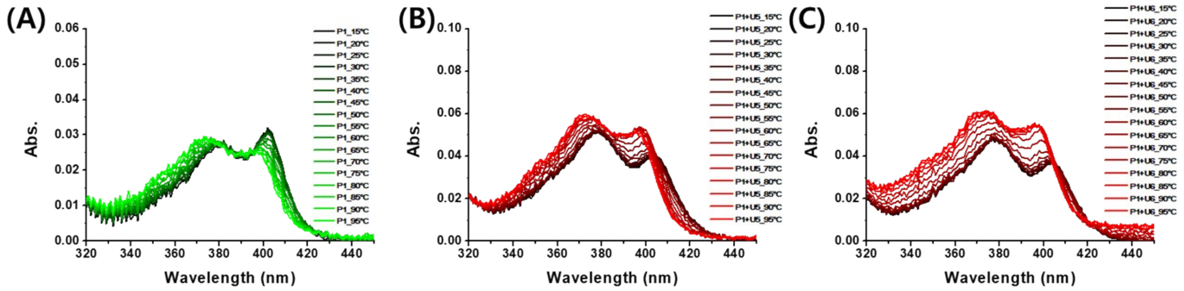

Figure S7. Fluorescence emission spectra of (A) single-stranded **P1** and **U5**–**U7** and (B–D) **P1** in the presence of a quencher strand **U5**–**U7** and **T19**

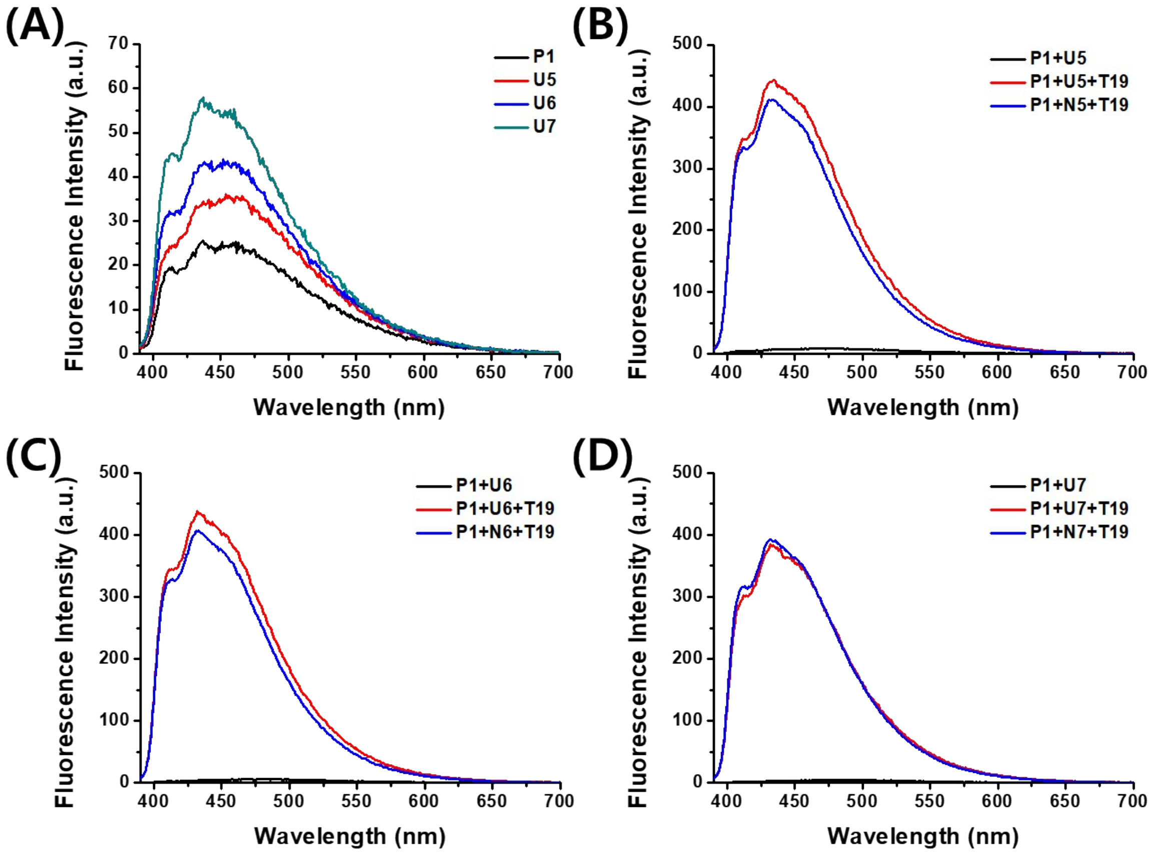

Figure S8. UV absorption spectra of **P1** in the presence of **Q5–Q7**

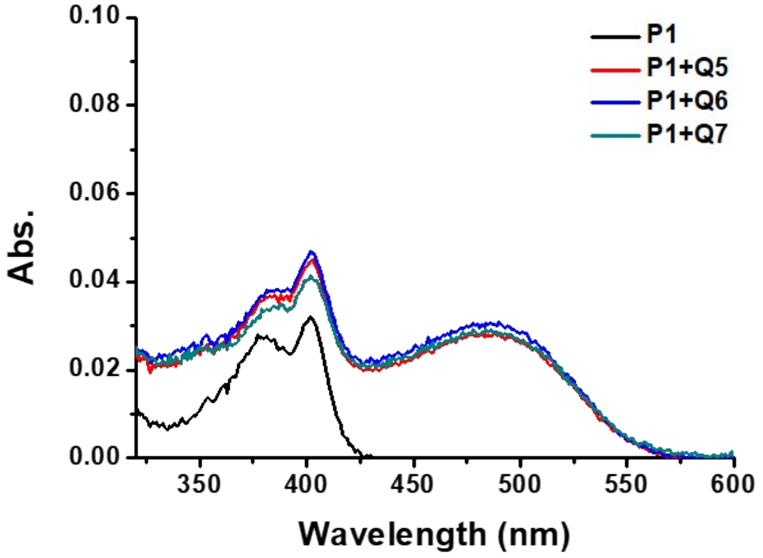

Figure S9. Fluorescence emission spectra of **P1** in the presence of **Q5–Q7** and **T19**

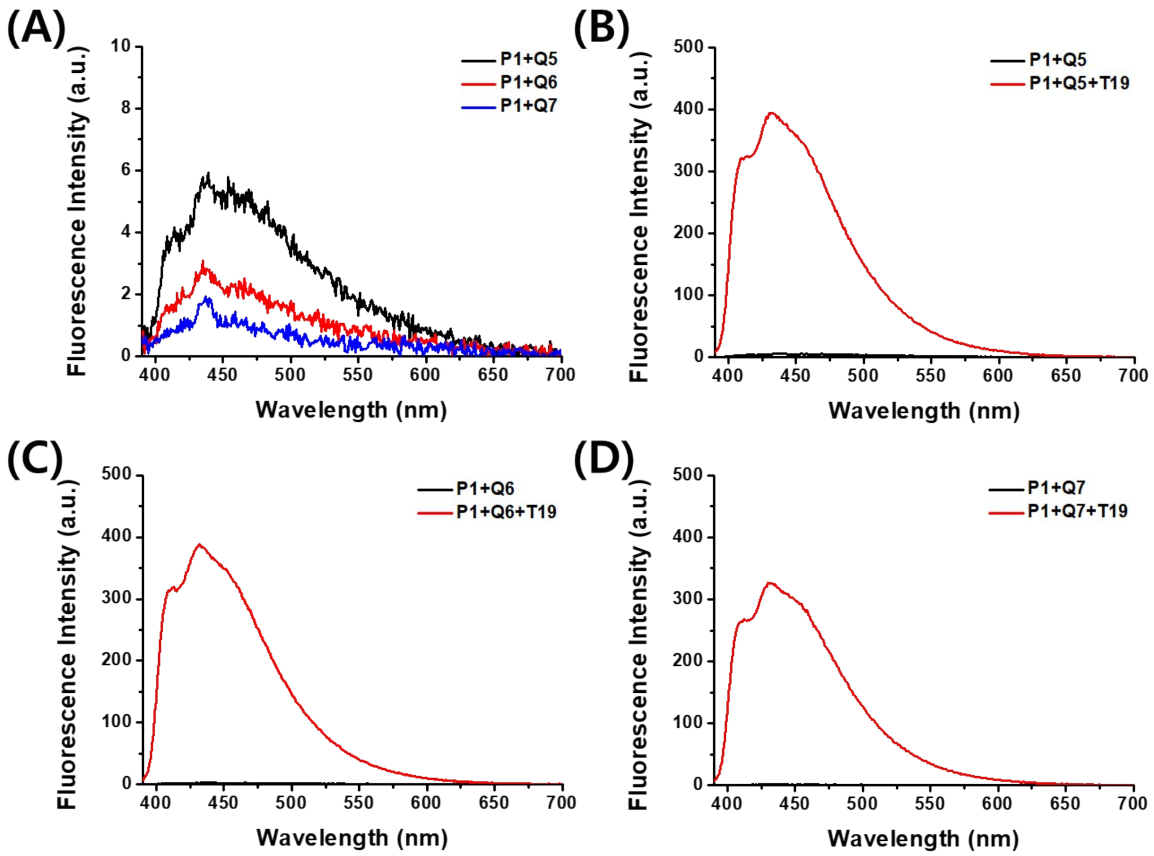

Figure S10. Melting curves of **P1** in the presence of (A) **U5–U7** and (B) **Q5–Q7**

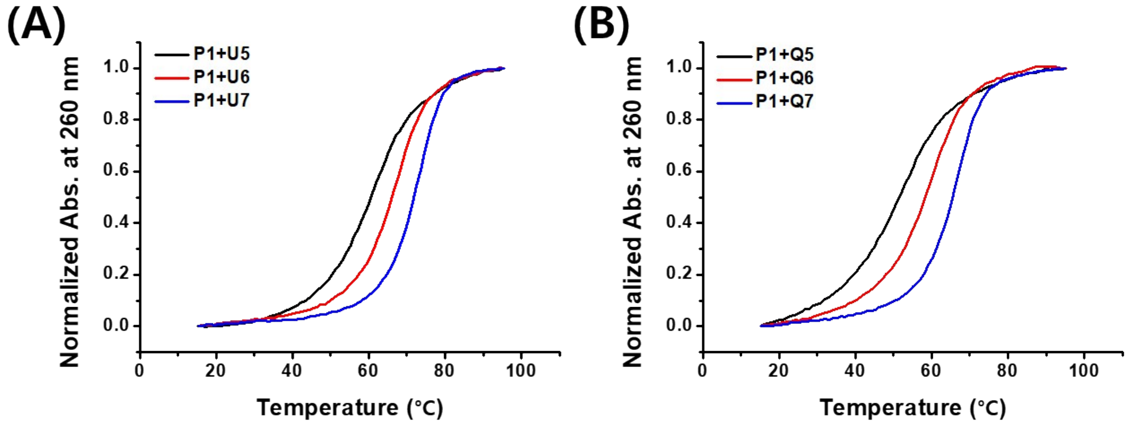

Table S5. Melting temperature of **P1** in the presence of **U5–U7** and **Q5–Q7**

Duplex	T_m (°C)
**P1 + U5**	60.1
**P1 + U6**	66.0
**P1 + U7**	71.8
**P1 + Q5**	51.5
**P1 + Q6**	58.2
**P1 + Q7**	65.3
**P1 + N5**	46.8
**P1 + N6**	56.0
**P1 + N7**	63.0
**PN + N5**	53.7
**PN + N6**	59.2
**PN + N7**	65.2

Figure S11. CD spectra of (A) **P1** and **T19** and (B–D) **P1** in the presence of a quencher strand **U5–U7** and **T19**

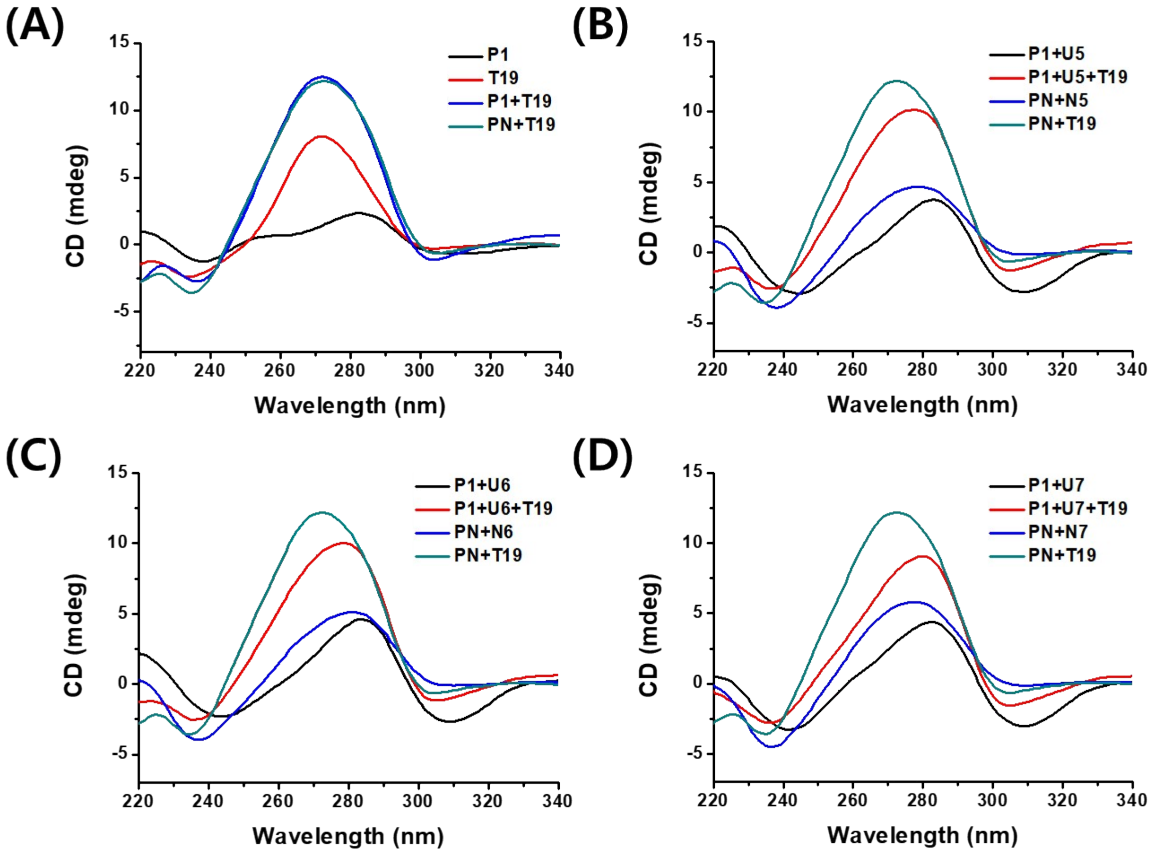

Figure S12. Native polyacrylamide gel electrophoresis (PAGE) images of **P1** with **T19** in the presence of **U5**–**U7** (A) stained with Stains-All and (B) under UV irradiation

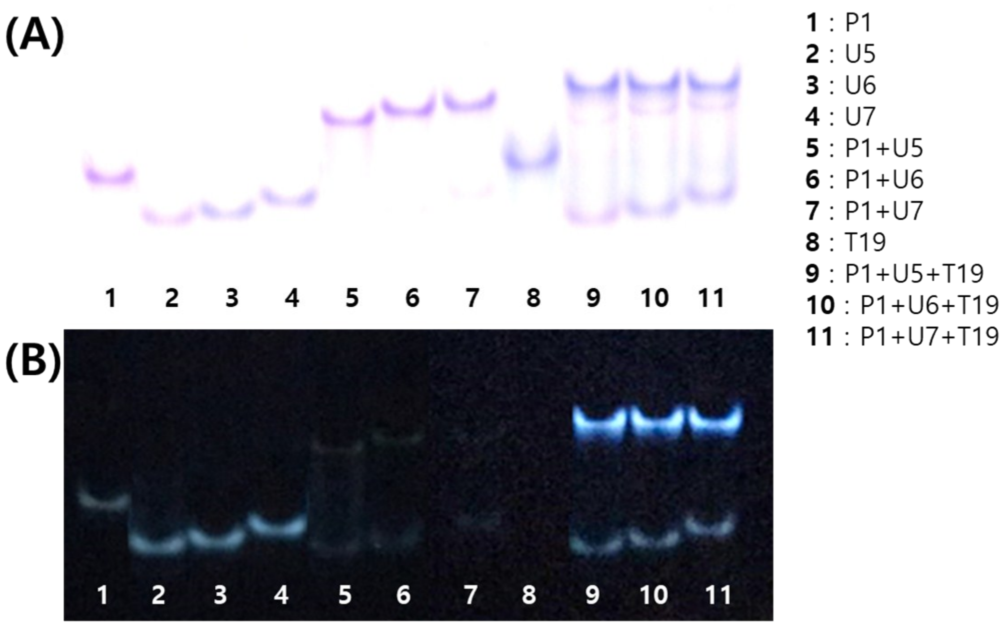

Figure S13. Time-dependent fluorescence intensity of **P1** in the presence of **U7** after the addition of **T19**

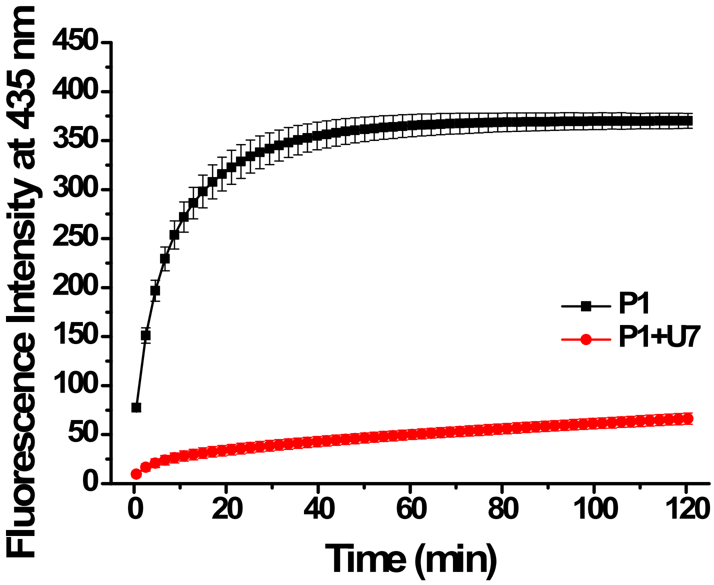

Supplement: RA-008-C7RA13349A-s001 [file RA-008-C7RA13349A-s001.pdf]
